# Extended analyses of rotavirus C (RVC) G-types and P-types reveal new cut-off value for the G-types and reclassification of strains

**DOI:** 10.1128/jvi.00049-25

**Published:** 2025-04-15

**Authors:** Belinda Euring, Maxi Harzer, Thomas W. Vahlenkamp

**Affiliations:** 1 Institute of Virology, Veterinary Faculty, University of Leipzig 9180, Leipzig, Germany; Emory University School of Medicine, Atlanta, Georgia, USA

**Keywords:** rotavirus, rotavirus group C, genotyping, sequence analysis, genetic diversity

## Abstract

Rotavirus C (RVC) is an important cause of gastroenteritis in humans and pigs and has also been detected in cattle, ferrets, minks, and dogs. Incidental zoonotic transmissions have been described. In contrast to rotavirus A (RVA), a complete genotyping system for RVC has not yet been established due to limited or incomplete sequence data. In this study, 138 complete nucleotide sequences for VP7 (G-type) and 97 complete nucleotide sequences for VP4 (P-type) of porcine RVC-positive samples have successfully been generated and genotyped. Together with available sequences from the NCBI database, phylogenetic analyses were conducted, cut-off values were re-evaluated, and the current classification system was adapted. Pairwise identity frequency analyses revealed a new cut-off value of 82% instead of the previous 85% for the G-type and confirmed the current cut-off value of 85% for the P-type. This resulted in the identification of 21 G-types and 39 P-types, including 4 new G-types and 10 new P-types. The results of the investigations expand the existing knowledge about the genetics of RVC and demonstrate the enormous diversity of porcine RVC sequences in particular.

**IMPORTANCE:**

This article provides a new sequence data set of porcine rotavirus C (RVC) strains. The extended full-length analysis of RVC G-types and P-types enabled us to review the current classification system. According to the guidelines of the rotavirus classification working group (RCWG), the results led to a new cut-off value of RVC G-types and required the reclassification of numerous RVC G-types. In addition, several new genotypes have been found. The present work closes the aforementioned knowledge gap and provides important, comprehensive data for RVC genetic diversity.

## INTRODUCTION

Rotaviruses are common enteric pathogens causing gastroenteritis in humans and in a variety of mammalian and avian species (1). Rotaviruses belong to the family *Sedoreoviridae* and are divided into nine species (*rotavirus alpha-* to *deltagastroenteritidis* and *phi-* to *jotagastroenteritidis*, formerly designated rotavirus A–D and F–J or RVA–RVD and RVF–RVJ, respectively) (2) based on the antigenic properties of their viral protein 6 (VP6) (3). Rotaviruses in common shrews have recently been described as putative novel rotavirus species K and L (4). RVA is the most extensively studied rotavirus species with high prevalence and clinical impact on humans and several other animal species (5–7). Over the last two decades, however, an increasing significance of rotavirus C (RVC), which so far could be detected in humans (8–12), pigs (13–21), cattle (22–24), dogs (25, 26), ferrets (27), and minks (28), was observed. There have been several outbreaks of RVC infections among children in Japan, South Korea, and India (10, 12, 29). In the latter, RVC was detected in 8.6% of outbreak cases, with severe disease observed in 70% of infected patients (9). An increasing trend in RVC infections can also be observed in animals (30), especially pigs (14, 21, 31, 32). Incidental porcine-to-human zoonotic transmissions and vice versa have been described (8, 33, 34). Several studies report a rise in prevalence with a higher detection rate in symptomatic than in asymptomatic animals (13, 14, 31). Higher RVC prevalences have been described in neonatal and suckling piglets (18, 35), as well as in weaners (36) or older pigs (37, 38). As RVC is also detected in asymptomatic animals (39), it is assumed that RVC continuously circulates in the pig holdings (40) resulting in a high genetic diversity of strains (18, 41, 42). This makes the pig the main reservoir for RVC and poses a major risk of zoonotic porcine-to-human transmissions (8, 40, 43).

The rotavirus particle consists of a triple-layered, non-enveloped capsid which encloses the viral genome of 11 genome segments of double-stranded RNA (dsRNA) (44). Those genome segments encode for six structural proteins (VP1, VP2, VP3, VP4, VP6, and VP7) and five or six non-structural proteins (NSP1-5/6). The innermost layer of the viral particle is formed by VP2. Attached to VP2 are complexes of VP1 (RNA-dependent RNA polymerase) and VP3 (methyltransferase). The middle layer of the particle consists of VP6, and the outermost layer of the viral particle is formed by VP7 and VP4. The latter two viral proteins can elicit neutralizing antibodies in the host and, therefore, represent the basis for the classification of rotaviruses into different genotypes, which is widely used for RVA (45–48). The dual genotyping system refers to the G-type (based on the nucleotide sequence of VP7, which is a glycoprotein) and P-type (based on the nucleotide sequence of VP4, which is protease-sensitive). Due to their immunological importance, VP7 and VP4 have been used for many years as targets for RVA vaccine development (49), highlighting the significance of the dual genotyping system (50). For RVA, the genotyping system has meanwhile been extended to all 11 genome segments (51, 52).

For RVC, efforts are going to contribute to the goal of obtaining a complete genotyping system similar to that for RVA (18, 37, 39, 41, 42, 53–56). A provisional genotyping system using all 11 genome segments has been proposed (41). Even though great progress has been made in RVC genotyping, there are still gaps to fill in order to understand genetic diversity and epidemiology. Therefore, continued genomic sequencing and comparative analyses are essential for developing a comprehensive genotyping framework.

To date, most of the sequence data for porcine RVC come from Japan, the USA, and South Korea (13, 15, 18, 41, 53, 54, 56). In Europe, only data from the Czech Republic, Ireland, and Italy are available (37, 39, 42, 57). In this study, a large number of complete sequences for VP4 and VP7 of European RVC strains from Germany, Austria, Denmark, Bulgaria, Slovakia, and the Netherlands were generated and phylogenetically analyzed in detail. To our knowledge, this is the first study that provides sequence information on RVC from the aforementioned countries.

## MATERIALS AND METHODS

### Samples

Fecal, swab, and gut samples from pigs that tested positive for RVC were used for molecular analyses. The samples were derived from diagnostic submission to the Institute of Virology, Faculty of Veterinary Medicine, Leipzig University, and to other veterinary diagnostic laboratories in Germany and Austria.

### RNA extraction

Prior to nucleic acid extraction, fecal samples were suspended in phosphate-buffered saline (PBS), fecal swabs were mounted in PBS, and gut samples were treated with a ball mill. RNA extraction was conducted using the Qiagen RNeasy Kit (Qiagen, Hilden, Germany) according to the manufacturer’s instructions. RNA was stored at −80°C until further use.

### RT-PCR

Two primer pairs were designed comprising the complete open reading frame (ORF) of the gene segment 9 (VP7) and gene segment 4 (VP4). Primer RVC VP7 forward (5′-AGCTGTCTGACAAACTGGTCTTCTTT-3′) binds completely within the 5′-untranslated region (UTR), and RVC VP7 reverse (5′- AGCCACATGATCTTGTTTACGCATACC-3′) overlaps partially with 11 base pairs from the 3′-UTR into the coding region. Primer RVC VP4 forward (5′-GATCRATGGCGTCYTCAC-3′) starts at the 5′-UTR and overlaps by 13 base pairs into the coding region. Primer RVC VP4 reverse (5′-GCCACATWWYAAGYYGRTCYCCTCA-3′) binds completely within the region of the 3′-UTR. If no PCR product for VP4 could be generated with the above-mentioned primer pair, primers published by Chepngeno et al. (14) were used. Reverse transcription (RT) was carried out in a two-step process using SuperScript III Reverse Transcriptase Kit (Invitrogen, Thermo Fisher Scientific, Waltham, USA). A mixture of primers (10 mM), dNTPs (10 mM), nuclease-free water, and RNA was heated to 97°C for 5 min and chilled on ice immediately for at least 1 min. A master mix containing buffer, reverse transcriptase (100 U), DTT (0.1 M), and Ribolock RNAse inhibitor (40 U) was subsequently added. RT was carried out at 55°C for 60 min, followed by heat inactivation of the reverse transcriptase at 70°C for 15 min. PCR was conducted using Q5 Hot Start High-Fidelity DNA Polymerase (New England Biolabs, Frankfurt, Germany) according to the manufacturer’s instructions. RT products were diluted 1:10 and used as templates. The amplification consisted of initial denaturation at 98°C for 30 s, followed by 35 cycles of 10 s of denaturation at 98°C, 30 s of annealing at 63°C for VP7 or at 61°C for VP4, and 30 s (VP7) or 2 min (VP4) elongation at 72°C, followed by a final elongation step for 2 min at 72°C. PCR products were analyzed on a 1%–1.5% agarose gel and purified using the GeneJET Gel Extraction Kit (Thermo Scientific, Waltham, USA) according to the manufacturer’s instructions.

### Sequence and phylogenetic analysis

**Fig 1:**
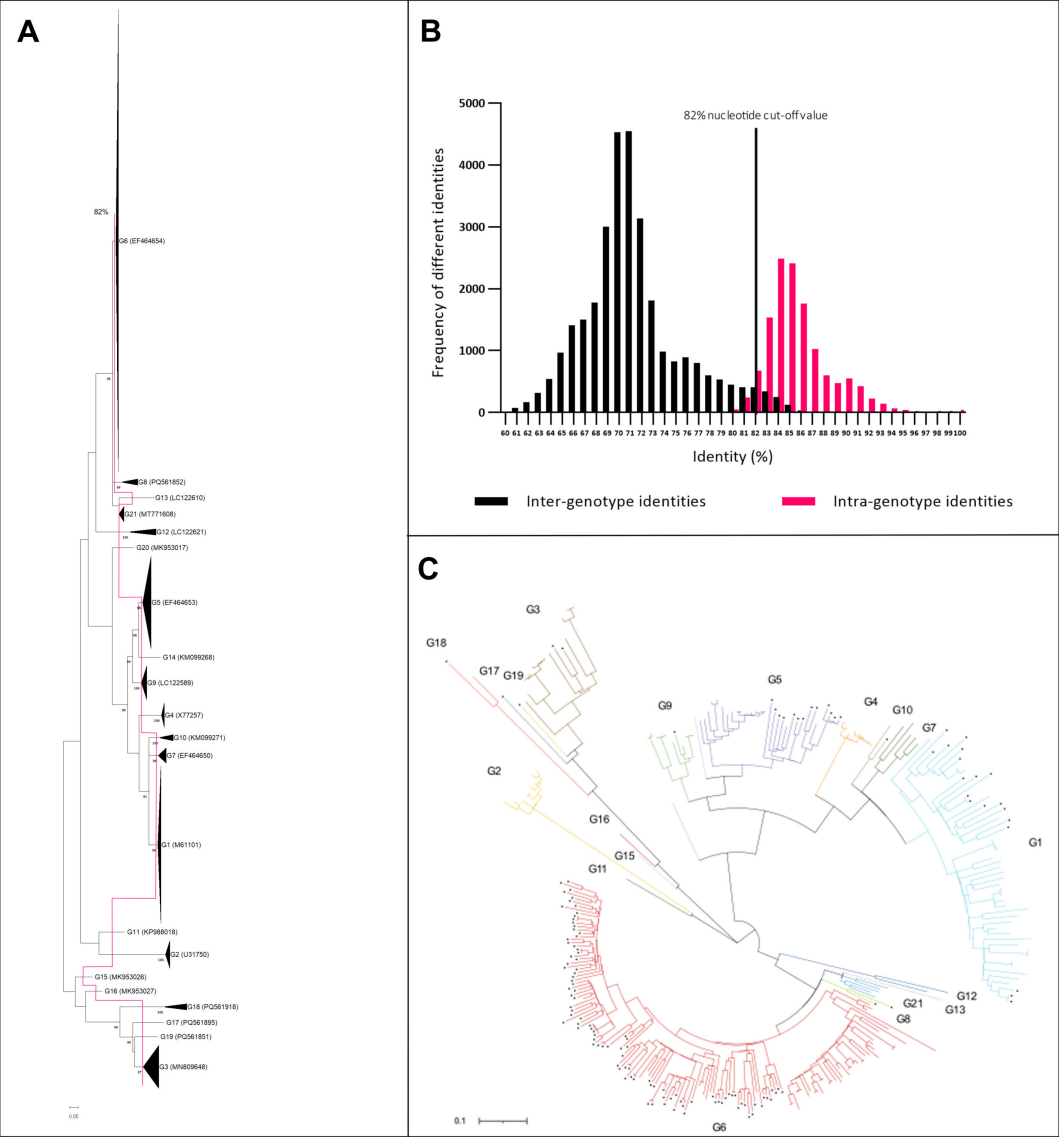
Phylogenetic analyses and pairwise identity frequency distribution graph for the G-type (VP7). (**A**) Phylogenetic tree based on the ORF nucleotide sequences of the VP7 gene (G-type) of RVC strains. The dendrogram was constructed in MegaX using Maximum Likelihood with General Time Reversible Model + G + I and 1,000 bootstrap replicates. Genotypes are represented by compressed clusters and designated on the right; the accession number of the respective reference strain is shown in brackets. The determined cut-off value of 82% is shown by the vertical line. (**B**) Nucleotide pairwise identity frequency graph of 295 complete VP7 ORF sequences. The proposed 82% nucleotide cut-off value is shown by the vertical line. (C) Phylogenetic tree based on the ORF nucleotide sequences of the VP7 gene (G-type) of RVC strains. The dendrogram was constructed in MegaX using Maximum Likelihood with General Time Reversible Model + G + I and 1,000 bootstrap replicates. The visualization of the tree was conducted using iTOL (58). Symbols (filled dots) indicate the sequences (*n* = 138) obtained in this study. Genotypes are specified with colored branches and associated designation outside of the tree.

**Fig 2:**
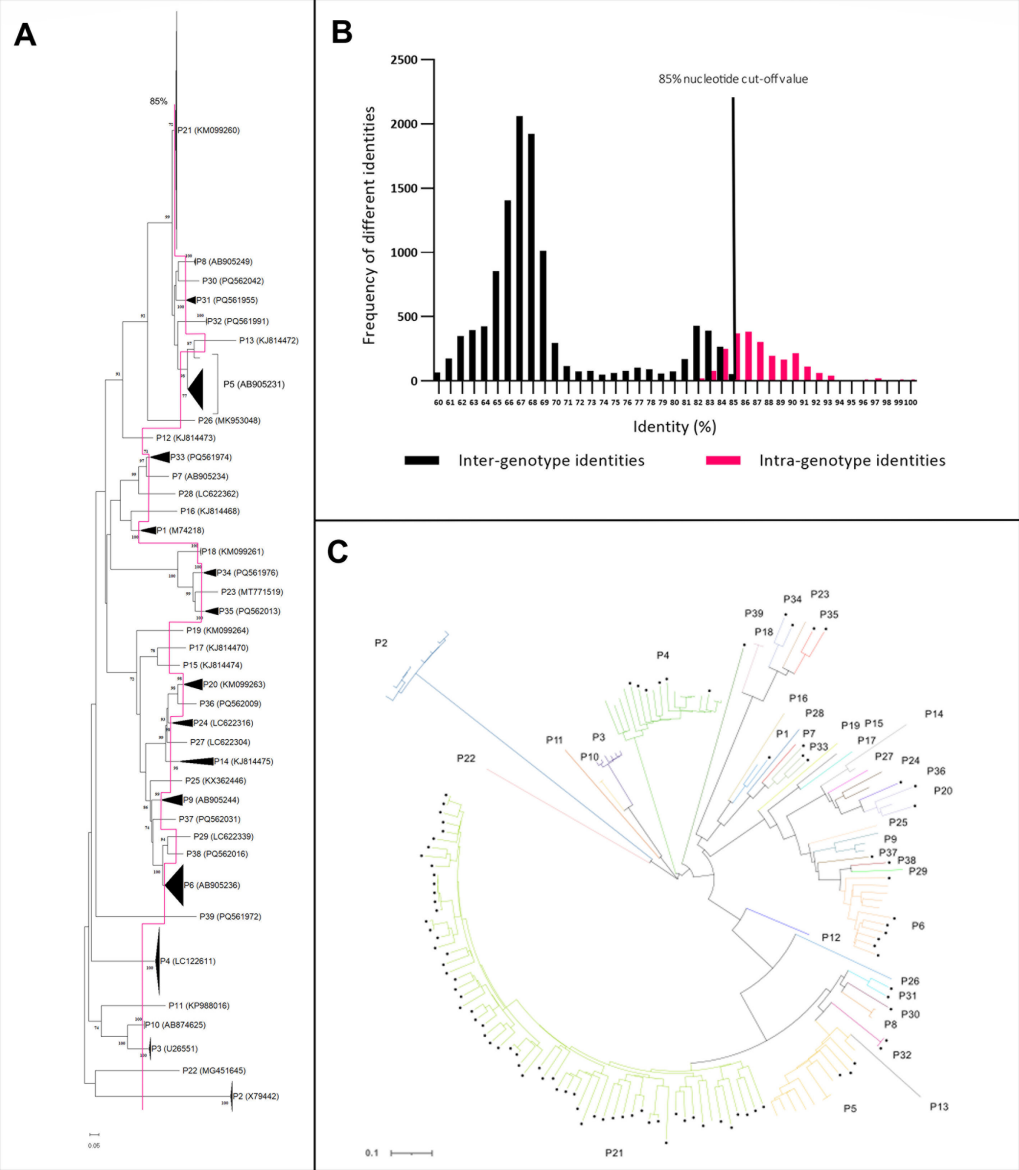
Phylogenetic analyses and pairwise identity frequency distribution graph for the P-type (VP4). (**A**) Phylogenetic tree based on the ORF nucleotide sequences of the VP4 gene (P-type) of RVC strains. The dendrogram was constructed in MegaX using Maximum Likelihood with General Time Reversible Model + G + I and 1,000 bootstrap replicates. Genotypes are represented by compressed clusters and designated on the right; the accession number of the respective reference strain is shown in brackets. The current cut-off value of 85% is shown by the vertical line. (**B**) Nucleotide pairwise identity frequency graph of 165 complete VP4 ORF sequences. The current 85% nucleotide cut-off value is shown by the vertical line. (**C**) Phylogenetic tree based on the ORF nucleotide sequences of the VP4 gene (P-type) of RVC strains. The dendrogram was constructed in MegaX using Maximum Likelihood with General Time Reversible Model + G + I and 1,000 bootstrap replicates. The visualization of the tree was conducted using iTOL (58). Symbols (filled dots) indicate the sequences (*n* = 97) obtained in this study. Genotypes are specified with colored branches and associated designation outside of the tree.

Purified PCR products were cloned into pJET1.2/Blunt vectors using the blunt-end protocol of the CloneJET PCR Cloning Kit (Thermo Scientific, Waltham, USA) and transformed into *Escherichia coli*. The transformation mixture was streaked out on agar plates containing ampicillin as a selective antibiotic. Plates were incubated for 16 h at 37°C. Colonies were used for PCR according to the manufacturer’s instructions. Clones carrying the respective insert were applied to overnight cultures using 10 mL LB (lysogeny broth) medium with 10 µL ampicillin. Cultures were incubated at 37°C and 220 rpm for 16 h. Plasmids were purified using the GenElute Plasmid Miniprep Kit (Sigma-Aldrich, St. Louis, USA) according to the manufacturer’s instructions and subsequently submitted for Sanger sequencing to Microsynth Seqlab Göttingen, Germany. Obtained sequences were aligned with the Clustal W method in MegaX software. Pairwise distances were calculated using Kimura-2-Parameter correction at the nucleotide level. Phylogenetic trees of the complete segment 9 and 4 sequences obtained in this study together with porcine, bovine, and human RVC strains available in the GenBank database were constructed in MegaX under Maximum Likelihood with General Time Reversible Model + G + I and 1,000 bootstrap replicates (Fig. 1A and 2A; Fig. S1 and S2). Visualization of the phylogenetic trees was conducted using iTOL (58) (Fig. 1C and 2C). Pairwise identity frequency graphs were constructed in GraphPad for VP7 and VP4 to obtain suitable cut-off values (Fig. 1B and 2B).

Overall, 138 complete sequences for VP7 and 97 complete sequences for VP4 were obtained and submitted to the NCBI database. A list with accession numbers and detailed phylogenetic trees can be found in the supplemental material (Fig. S1 and S2; Tables S1 and S2).

## RESULTS

Phylogenetic trees were evaluated under orientation at the proposed cut-off value of 85% for the G-type and the P-type (41). Different alternatives were investigated by comparing inter-genotype and intra-genotype identities to assign certain clusters as different genotypes. To examine if the proposed cut-off value of 85% is still applicable, pairwise identity frequency graphs on the nucleotide level were constructed as described by Matthijnssens et al. (51, 52). The analyses confirmed the established cut-off value of 85% for the P-type and revealed a new cut-off value of 82% for the G-type (see Fig. 1B and 2B). The phylogenetic analyses revealed a total of 21 G-types and 39 P-types.

Of the 138 sequences for VP7, 58 sequences had an ORF length of 999 nucleotides (nt) and 80 sequences had an ORF length of 1,011 nt. Of the 97 sequences for VP4, 76 sequences had an ORF length of 2,199 nt, 6 had an ORF length of 2,217 nt, 4 sequences each had an ORF length of 2,211 nt, 2,208 nt, and 2,190 nt, and 1 sequence each had an ORF length of 2,205 nt, 2,202 nt, and 2,214 nt.

### Detailed description of selected G-types (VP7)

#### G1

**Fig 3:**
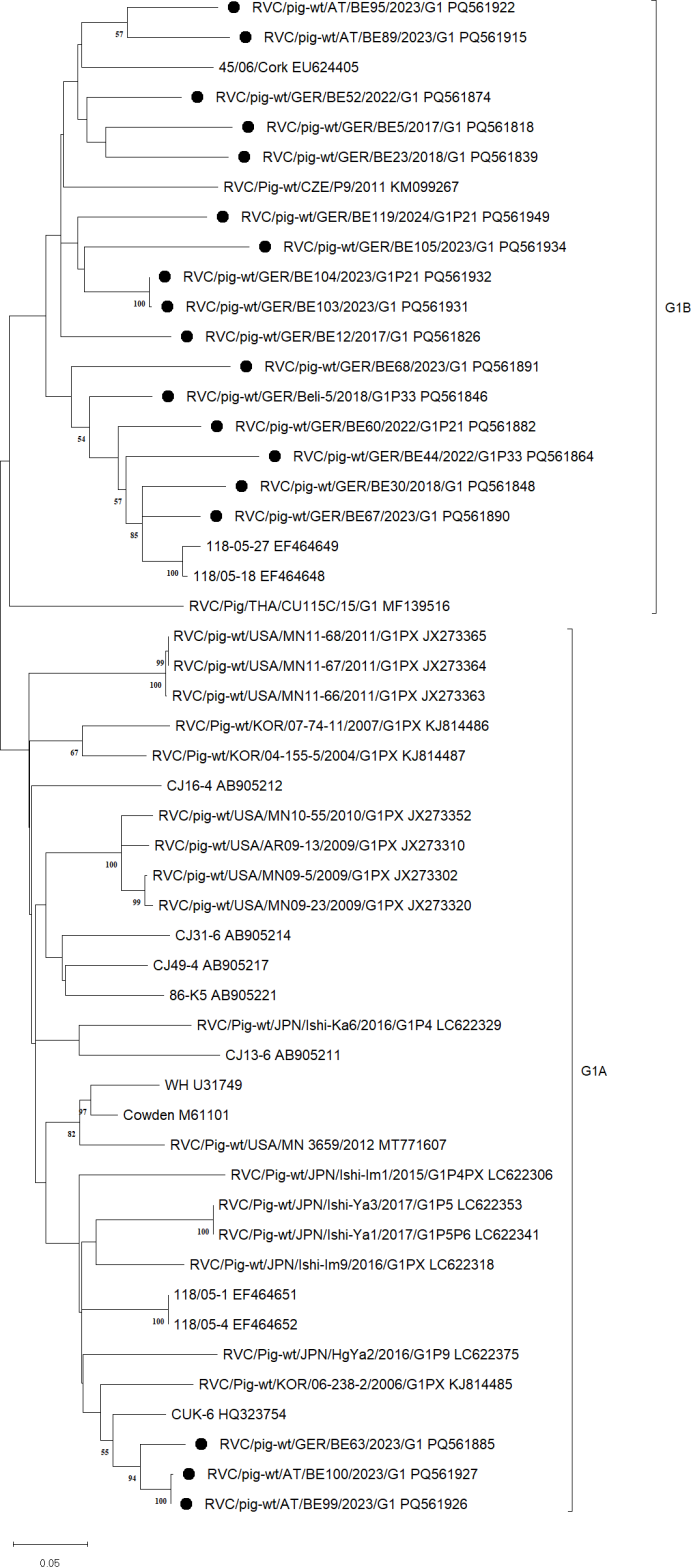
Subtree of cluster G1 displayed separately from the detailed phylogenetic tree of VP7 (Fig. S1). The dendrogram was constructed in MegaX using Maximum Likelihood with General Time Reversible Model + G + I and 1,000 bootstrap replicates. Symbols (filled dots) indicate the sequences obtained in this study. Subtypes are specified with brackets and associated designation on the right.

The dendrogram for G1 shows a division of the sequences into two subclusters, named G1A and G1B (see Fig. 3). The pairwise distances in subcluster G1A range from 82% to 100%, whereas the identities between G1A and G1B cover an area from 79% to 89%. Pairwise distances within subcluster G1B range from 81% to 100%. As there are several sequences between G1A and G1B with identities that are well above the newly determined cut-off value of 82%, the two subclusters cannot be divided into two distinct genotypes. Moreover, sequences which had been previously classified into genotypes G25 (MF139516.1, partial), G26 (KM099267.1), G27 (EU624405.1, partial), and G28 (EF464649.1) clustered into G1 and therefore had been reclassified as G1.

#### G3

Based on the new cut-off value of 82%, sequences KJ814488.1 and KM099266.1, which had been previously classified into genotypes G18 and G16, respectively, now belong to genotype G3. Sequences LC622364.1, LC622387.1, and LC622398.1, which had been previously classified as genotype G32, also clustered into G3 and were therefore reclassified as G3.

#### G5

**Fig 4:**
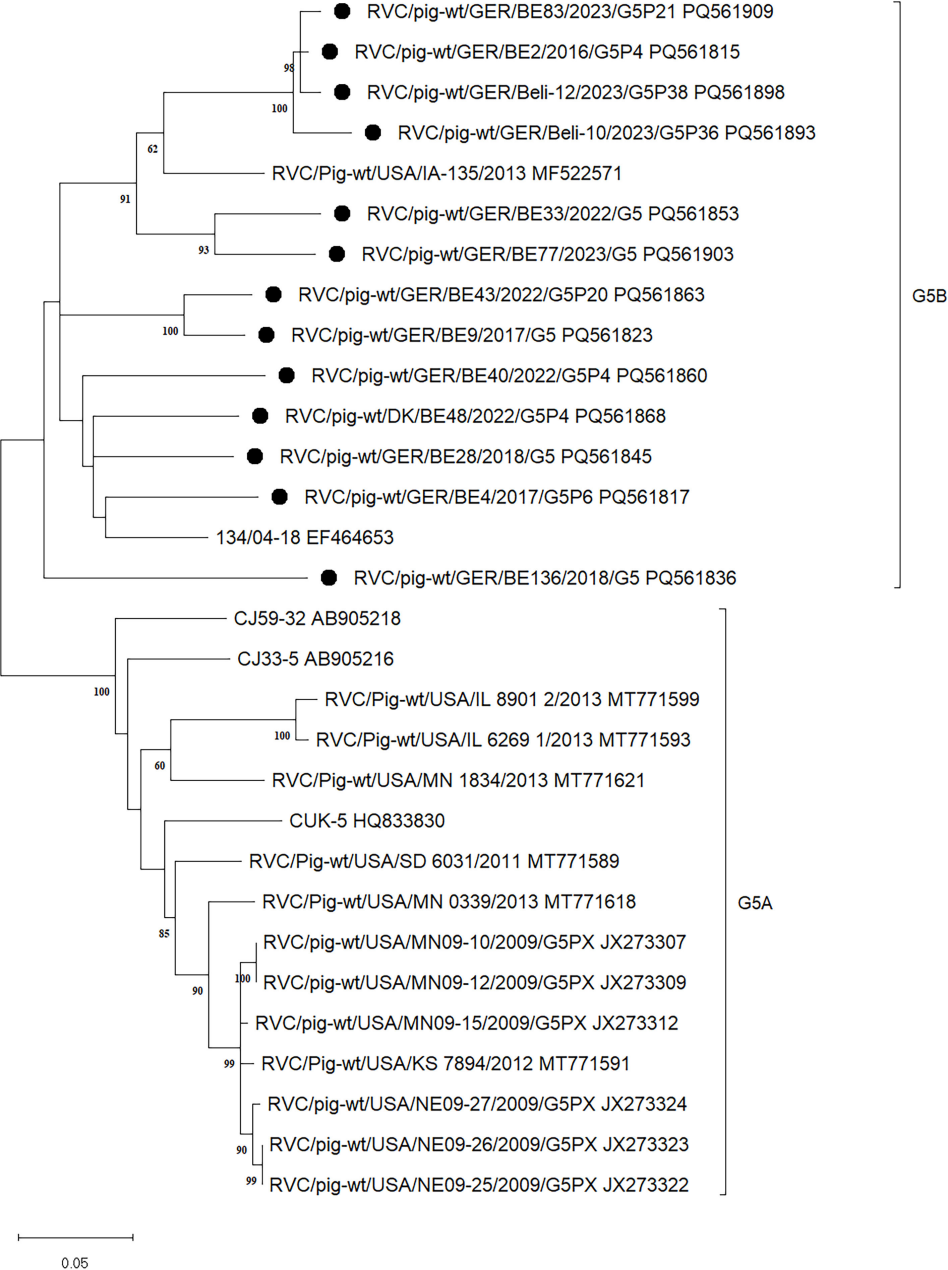
Subtree of cluster G5 displayed separately from the detailed phylogenetic tree of VP7 (Fig. S1). The dendrogram was constructed in MegaX using Maximum Likelihood with General Time Reversible Model + G + I and 1,000 bootstrap replicates. Symbols (filled dots) indicate the sequences obtained in this study. Subtypes are specified with brackets and associated designation on the right.

This cluster can be divided into two subclusters (see Fig. 4): the lower subcluster, here referred to as G5A, consists of sequences that were all previously classified as G5 and the upper subcluster, here referred to as G5B, contains one sequence which had been previously classified as G5, and one sequence which had been previously classified as G24 (MF522571.1). The identities within G5A reach from 88% to 100%, whereas the identities within G5B range from 83% to 98% and the identities between G5A and G5B range from 81% to 87%. As the identities between the subclusters are almost all above the cut-off value of 82%, the subclusters cannot form separate genotypes. Therefore, the genotype previously identified as G24 was reclassified as G5.

#### G6

**Fig 5:**
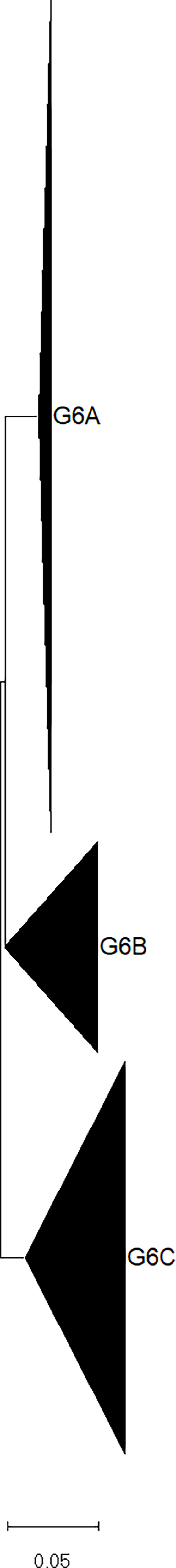
Subtree with compressed subclusters of cluster G6 displayed separately from the detailed phylogenetic tree of VP7 (Fig. S1). The dendrogram was constructed in MegaX using Maximum Likelihood with General Time Reversible Model + G + I and 1,000 bootstrap replicates. Subtypes are designated on the right.

This cluster displays a division into three subclusters, described as G6A, G6B, and G6C (see Fig. 5). The distribution of pairwise identities is like G1, where a wide range of identities within and between the subclusters can be seen, but also a lot of identities between the subclusters lying well above the proposed cut-off value. For that reason, there is no clear division of the subclusters, and therefore they cannot be assigned as distinct genotypes. The identities within G6A and G6B range from 81% to 100%, whereas the identities within G6C range from 77% to 100%. The distribution of pairwise identities between the subclusters ranges from 76% to 88%, so that the overall spread width goes from 76% to 100% in cluster G6. Moreover, sequences that had previously been classified as G19 (MF522750.1), G20 (AB905222.1), and G8 (EU624403.1) clustered into G6 (see Fig. S1). Therefore, these sequences were reclassified as G6.

#### G7

Sequences previously classified as G7 (EF464650.1), G15 (KM099265.1), and G17 (KJ814502.1, KJ814504.1, KJ814505.1) all form one cluster. Identities within this cluster range from 84% to 93%. As this is well above the new cut-off value of 82%, the aforementioned sequences were put together and reclassified as G7.

#### G12

The sequence previously classified as G22 (KX825945.1) clusters together with G12 (LC122621.1). The identity between these two sequences is 83%. Therefore, G22 has been reclassified as G12.

Four new G-types were determined in this study, which were assigned as G8 and G17–G19 (Table 2).

Since the cut-off value of 82% led to changes in the classification of the established genotypes, the G-types were reorganized for better clarity. The new categorization proposed here can be found in Tables 1 and 2.

**TABLE 1:** Reclassification of RVC G-types

Accession no.	References	Formerly classified as	Classified as
EU624403.1	(39, 41)	G8	G6
KM099265.1	(41, 42)	G15	G7
KM099266.1	(41, 42)	G16	G3
KJ814502.1 KJ814504.1 KJ814505.1	(41, 59)	G17	G7
KJ814488.1	(41, 59)	G18	G3
MF522750.1	(56)	G19	G6
AB905222.1	(56, 60)	G20	G6
KX825945.1	(56)	G22	G12
MF522571.1	(56)	G24	G5
MF139516.1	(56)	G25	G1
KM099267.1	(42, 56)	G26	G1
EU624405.1	(39, 56)	G27	G1
EF464649.1	(56, 57)	G28	G1
LC622398.1 LC622364.1 LC622387.1	(55)	G32	G3

**TABLE 2:** New nomenclature of RVC G-types

Former G-type	New G-type	Reference sequence (accession no.)	Reference
G1, G25, G26, G27, G28	G1	M61101.1	–^*a*^
G2	G2	U31750.1	(61)
G3, G16, G18, G32	G3	MN809648.1	(62)
G4	G4	X77257.1	–
G5, G24	G5	EF464653.1	(57)
G6, G8, G19, G20	G6	EF464654.1	(57)
G7, G15, G17	G7	EF464650.1	(57)
GW	G8	PQ561852.1	This study
G9	G9	LC122589.1	(54)
G10	G10	KM099271.1	(42)
G11	G11	KP988018.1	(26)
G12, G22	G12	LC122621.1	(54)
G13	G13	LC122610.1	(54)
G14	G14	KM099268.1	(42)
G30	G15	MK953026.1	(63)
G29	G16	MK953027.1	(63)
GY	G17	PQ561895.1	This study
G31, GX	G18	PQ561918.1	This study
GZ	G19	PQ561851.1	This study
G23	G20	MK953017.1	(63)
G21	G21	MT771608.1	(56)

^*a*^ –, no reference provided.

### P-type (VP4)

**Fig 6:**
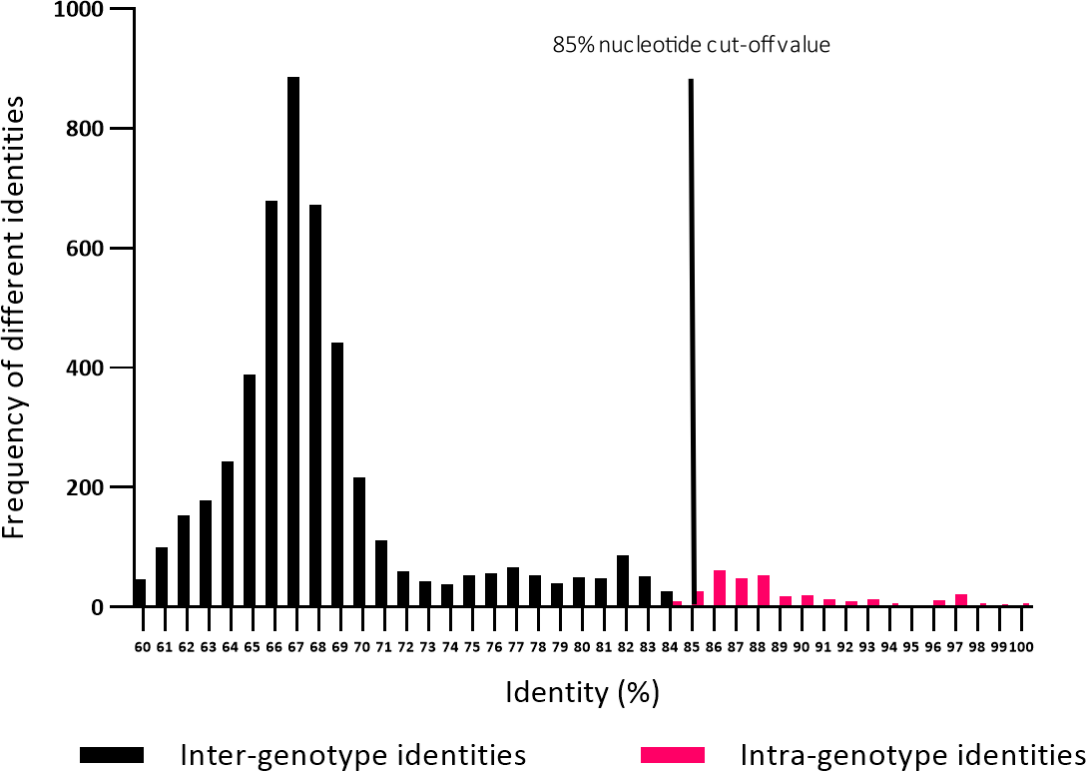
Nucleotide pairwise identity frequency graph of 102 complete VP4 ORF sequences without the genotype P21. The current 85% nucleotide cut-off value is shown by the vertical line.

Evaluation of the phylogenetic tree for VP4 revealed great consistency with previously published results of classification (41). There is only one cluster, P21, which shows a wider range of identities, comparable with G6 from the G-types. The pairwise identities within P21 range from 81% to 100%, whereas identities within the other P-types do not fall below 85%, except for P33, where an identity of 84.6% can be found. As can be seen in Fig. 6, P21 alone is responsible for the deviations in frequency distribution.

In this study, 10 new P-types could be found, which were assigned as P30–P39. A complete overview of currently existing P-types can be found in Table 3.

**TABLE 3:** Classification of P-types

Former P-type	New P-type	Reference sequence (accession no.)	Reference
P1	P1	M74218.1	–^*b*^
P2	P2	X79442.1	–
P3	P3	U26551.1	–
P4	P4	LC122611.1	(54)
P5	P5	AB905231.1	(60)
P6	P6	AB905236.1	(60)
P7	P7	AB905234.1	(60)
P8	P8	AB905249.1	(60)
P9	P9	AB905244.1	(60)
P10	P10	AB874625.1	(23)
P11	P11	KP988016.1	(26)
P12	P12	KJ814473.1	(59)
P13	P13	KJ814472.1	(59)
P14	P14	KJ814475.1	(59)
P15	P15	KJ814474.1	(59)
P16	P16	KJ814468.1	(59)
P17	P17	KJ814470.1	(59)
P18	P18	KM099261.1	(42)
P19	P19	KM099264.1	(42)
P20	P20	KM099263.1	(42)
P21	P21	KM099260.1	(42)
P22	P22^*a*^	MG451645.1 ^*a*^	(41)
P23	P23	MT771519.1	(56)
P24	P24	LC622316.1	(55)
P25	P25	KX362446.1	(64)
P26	P26	MK953048.1	(63)
P27	P27	LC622304.1	(55)
P28	P28	LC622362.1	(55)
P6	P29	LC622339.1	(55)
PQ	P30	PQ562042.1	This study
PR	P31	PQ561955.1	This study
PS	P32	PQ561991.1	This study
PT	P33	PQ561974.1	This study
PU	P34	PQ561976.1	This study
PV	P35	PQ562013.1	This study
PW	P36	PQ562009.1	This study
PX	P37	PQ562031.1	This study
PY	P38	PQ562016.1	This study
PZ	P39	PQ561972.1	This study

^*a*^ This sequence is a partial sequence. In future, a complete sequence should be found that can serve as a reference sequence.

^*b*^ –, no reference provided.

## DISCUSSION

The dual genotyping system based on G-types and P-types has been used successfully for many years to classify rotaviruses, especially RVA, into different genotypes. Due to their immunological importance also for RVC, a similar dual genotyping model is in place. For RVC, however, sequence data were limited due to a relatively low number of deposited complete genome segments for the ORF 9 and 4. Several deposited RVC genotypes are based on partial sequence information, which restricts reliability. With the complete gene segment 9 and 4 sequences provided and analyzed in this study, a new basis for the classification of RVC strains is provided. The divergence of the pairwise distances for several G-type clusters was found to lie beyond the previously postulated cut-off value of 85%. This mainly concerns G1, G3, G5, and G6, which all consist of porcine sequences. This demonstrates the enormous genetic diversity of porcine RVCs. No meaningful clusters due to high deviation of the pairwise identities could be obtained with the previous cut-off value of 85%. The frequency distribution graph based on these analyses clearly shows a downward shift of the cut-off value to 82% (Fig. 1B). It is possible that with the acquisition of new sequence data in future studies, the cut-off value will decrease even further and reach a level similar to that of RVA (51, 52). This is particularly evident when looking at the clusters from G6 to G21 (see Fig. 1A and C; Fig. S1). The sequences of G8 and G21 have both very low and in some cases relatively high identities (79.1%–86.4% for G8 and 78.2%–90.4% for G21) to some sequences from G6. However, the branching of the phylogenetic tree shows a clear separation of the G8 and G21 clusters from G6. In addition, G21 is further separated from G6 by the sequence of G13, which has only very low identities with the sequences from G6 (78.0%–82.2%). If more sequence data become available in the future, it is possible that the branches will be separated differently and that G8 and G21 (possibly even G13) will cluster into G6. G6 forms the largest cluster, which diverges quite widely in its pairwise identities. We achieved the best separation in the pairwise distances frequency distribution with G6 as genotype and G6A, G6B, and G6C as subclusters, which do not represent independent genotypes (Fig. 5).

When looking at G5 and G1, the formation of two subclusters can be seen, G5A and G5B (Fig. 4) as well as G1A and G1B (Fig. 3). Due to the fact that the pairwise identities between the subclusters show a high divergence both in the lower and higher ranges, these could not be separated as independent genotypes. A separation would not have led to a meaningful division of the genotypes as a whole. The phylogenetic tree of G3 splits up strongly. The sequences LC622387.1, LC622364.1, and LC622398.1 are above the postulated cut-off value of 82% only in comparison with just two other sequences from the same cluster. Nevertheless, they cannot be separated from G3 due to the topology of the tree. With more sequence data in the future, the above-mentioned sequences will either cluster more clearly into G3 or will be even more separated from it. Due to the new cut-off value, some sequences that were already labeled as specific genotypes were reclassified and partially integrated into existing genotypes. This concerns G1, G3, G5, and G6, as well as G7 and G12 (see Table 1). The new classification was carried out in accordance with the data analyzed in this study. In order to improve the clarity of all existing genotypes, some have been renamed, as shown in Table 2. Four new genotypes were also identified in this study, which were categorized as G16, G17, G18, and G8. The pairwise distances of G16 to the remaining genotypes were a maximum of 80.6%, those of G17 81.4%, and those of G18 75.0%. Thus, these three sequences form separate genotypes. With regard to G8, the situation is as described above; although the pairwise distances to G6 go up to 86.4%, the sequences can be separated as an independent genotype due to the tree topology and the low remaining distances, which represent the larger proportion.

With regard to the P-types, most of the findings were in concordance with previously published results (41, 54). The frequency distribution graph of the pairwise distances confirmed the current cut-off value of 85% (Fig. 2B). In contrast to the G-types, only one genotype is responsible for overlaps in the frequency distribution graph, namely P21. This genotype forms the largest of the P-types (Fig. 2A and C) with the widest divergence of the pairwise distances of the containing sequences. Without this cluster, there would be an almost perfect separation of the genotypes with a cut-off value of 85% (see Fig. 6). It is possible that with more sequence data, greater dispersion will also occur for other genotypes, and the picture will approach that of the G-types. Only one sequence had to be reclassified as P29, which was formerly categorized as P6 by Oki et al. (55) but has only pairwise identities of 81.6%–83.6% to the sequences from P6 and is separated from this cluster by a sequence identified in this study, which was categorized as P38. Another feature is represented by P13 and one sequence from P5 (KJ814469.1). In the phylogenetic tree, these two sequences originate from the same node, while the other sequences from the P5 cluster are separated from them by arising from another node. Although KJ814469.1 has an identity of 83.6% to P13, this is still below the cut-off value of 85%, and the identities of P13 to the remaining sequences from P5 amount to a maximum of only 78.9%. Therefore, P13 must be considered a separate genotype. KJ814469.1, however, has an identity of up to 88.2% to the other P5 sequences and therefore cannot be separated from P5, although the branching of the tree would suggest this (see Fig. 2A and C; Fig. S2). Additionally, 10 new P-types were found with pairwise identities below or on the edge of 85% to the other genotypes (a maximum of 84.6% for P30, 85.2% for P31, 82.8% for P32, 83.3% for P33, 84.0% for P34, 84.1% for P35, 85.6% for P36, 83.0% for P37, 85.2% for P38, and only 69.2% for P39). The genotypes with a maximum slightly above 85% are either separated by other genotypes from the sequences with which they have such an identity or have significantly lower further pairwise identities to other sequences from the same affected cluster. Therefore, these sequences were also categorized as independent genotypes.

In the future, it would be desirable to obtain even more sequence data of different RVC strains from the remaining gene segments and also from other animal species. This would also enable investigations to address recombinations, reassortment events, and interspecies transmissions (33, 43, 54, 55, 65–67).

The porcine RVC sequences analyzed in this study were grouped into a total of 10 G-types and 16 P-types. In total, porcine sequences account for 18 of 21 G-types and 35 of 39 P-types. This demonstrates their high diversity and importance for the evolution of RVC with the pig as a reservoir host. The data generated in this study led to a reclassification of the G-types by an adjusted cut-off value and resulted in the addition of four new G-types and 10 new P-types contributing to a detailed insight into the pronounced genetic diversity of RVC.

## Data Availability

Nucleotide sequences generated in this study were deposited at the NCBI database (https://www.ncbi.nlm.nih.gov/nucleotide/) and can be accessed through accession numbers PQ561814–PQ561950 and PQ606076 (see Tables S1 and S2).

## SUPPLEMENTAL MATERIAL

Fig. S1 and S2; Tables S1 and S2 jvi.00049-25-s0001.pdf Phylogenetic trees and nucleotide sequences with associated accession numbers.

Table S3 jvi.00049-25-s0002.xlsx Intragenotype identity G-type.

Table S4 jvi.00049-25-s0003.xlsx Intragenotype identity P-type.

Table S5 jvi.00049-25-s0004.xlsx Pairwise identities.

ASM does not own the copyrights to Supplemental Material that may be linked to, or accessed through, an article. The authors have granted ASM a non-exclusive, world-wide license to publish the Supplemental Material files. Please contact the corresponding author directly for reuse.
