# Supplementary material for: Extended analyses of rotavirus C (RVC) G-types and P-types reveal new cut-off value for the G-types and reclassification of strains
Source: J Virol. 2025 Apr 15;99(5):e00049-25. doi: 10.1128/jvi.00049-25 (PMC12090754; doi:10.1128/jvi.00049-25)
Supplement: Fig. S1 and S2; Tables S1 and S2 — Phylogenetic trees and nucleotide sequences with associated accession numbers. [file jvi.00049-25-s0001.pdf]

SUPPLEMENTAL MATERIAL

FIGURES:

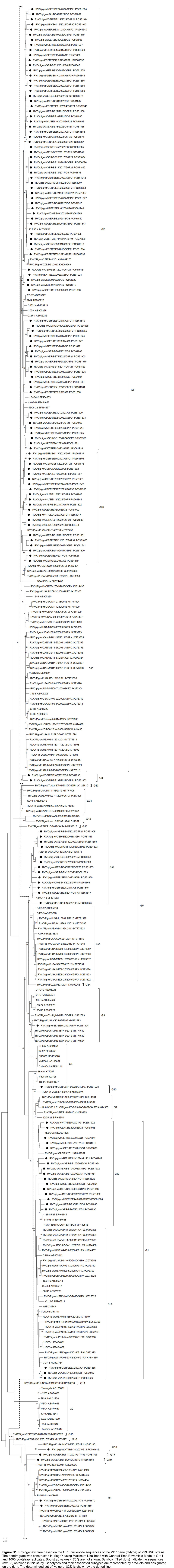

Figure S1. Phylogenetic tree based on the ORF nucleotide sequences of the VP7 gene (G-type) of 299 RVC strains. The dendrogram was constructed in MegaX using Maximum Likelihood with General Time Reversible Model + G + I and 1000 bootstrap replicates. Bootstrap values < 70% are not shown. Symbols (filled dots) indicate the sequences (n=138) obtained in this study. Genotypes and their associated subtypes are represented by brackets and designated on the right. The determinded cut-off value of 82% is shown by the dotted line.

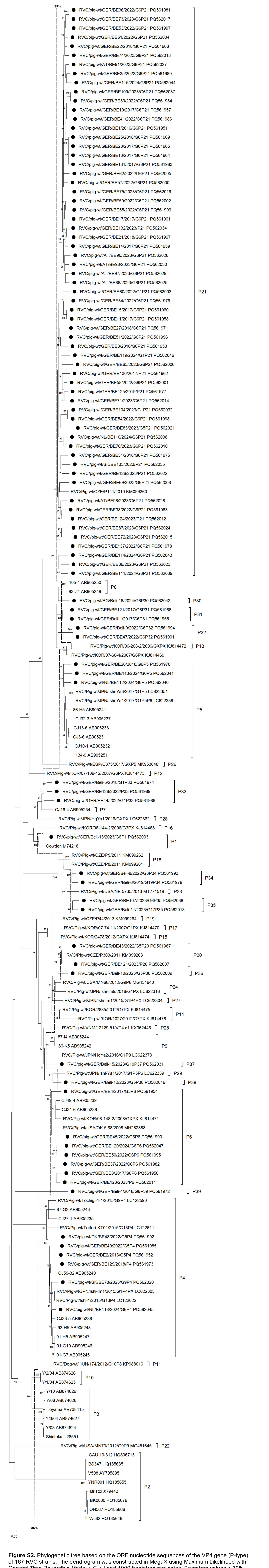

Figure S2. Phylogenetic tree based on the ORF nucleotide sequences of the VP4 gene (P-type) of 167 RVC strains. The dendrogram was constructed in MegaX using Maximum Likelihood with General Time Reversible Model + G + I and 1000 bootstrap replicates. Bootstrap values < 70% are not shown. Symbols (filled dots) indicate the sequences (n=97) obtained in this study. Genotypes are represented by brackets and designated on the right. The current cut-off value of 85% is shown by the dotted line.

TABLES:

Strain name (G-type)	NCBI-Accession-Number
RVC/pig-wt/GER/BE1/2016/G6P21	PQ561814
RVC/pig-wt/GER/BE2/2016/G5P4	PQ561815
RVC/pig-wt/GER/BE3/2016/G6P21	PQ561816
RVC/pig-wt/GER/BE4/2017/G5P6	PQ561817
RVC/pig-wt/GER/BE5/2017/G1	PQ561818
RVC/pig-wt/GER/BE6/2017/G6	PQ561819
RVC/pig-wt/GER/Beli-1/2017/G6P31	PQ561820
RVC/pig-wt/GER/BE7/2017/G6	PQ561821
RVC/pig-wt/GER/BE8/2017/G6P6	PQ561822
RVC/pig-wt/GER/BE9/2017/G5	PQ561823
RVC/pig-wt/GER/BE10/2017/G6P21	PQ561824
RVC/pig-wt/GER/BE11/2017/G6P21	PQ561825
RVC/pig-wt/GER/BE12/2017/G1	PQ561826
RVC/pig-wt/GER/BE13/2017/G6	PQ561827
RVC/pig-wt/GER/BE14/2017/G6P21	PQ561828
RVC/pig-wt/GER/BE15/2017/G6P21	PQ561829
RVC/pig-wt/GER/BE16/2017/G6	PQ561830
RVC/pig-wt/GER/BE17/2017/G6P21	PQ561831
RVC/pig-wt/GER/BE18/2017/G6P21	PQ561832
RVC/pig-wt/GER/BE19/2017/G6	PQ561833
RVC/pig-wt/GER/BE20/2017/G6P21	PQ561834
RVC/pig-wt/GER/BE121/2017/G6P31	PQ561835
RVC/pig-wt/GER/BE136/2018/G5	PQ561836
RVC/pig-wt/GER/BE21/2018/G6P21	PQ561837
RVC/pig-wt/GER/BE22/2018/G6P21	PQ561838
RVC/pig-wt/GER/BE23/2018/G1	PQ561839
RVC/pig-wt/GER/BE24/2018/G6	PQ561840
RVC/pig-wt/GER/BE25/2018/G6P21	PQ561841
RVC/pig-wt/GER/BE26/2018/G6P5	PQ561842
RVC/pig-wt/GER/BE27/2018/G6P21	PQ561843
RVC/pig-wt/GER/Beli-4/2018/G6P39	PQ561844
RVC/pig-wt/GER/BE28/2018/G5	PQ561845
RVC/pig-wt/GER/Beli-5/2018/G1P33	PQ561846
RVC/pig-wt/GER/BE29/2018/G6	PQ561847
RVC/pig-wt/GER/BE30/2018/G1	PQ561848
RVC/pig-wt/GER/BE31/2018/G6P21	PQ561849
RVC/pig-wt/GER/BE32/2019/G6	PQ561850
RVC/pig-wt/GER/Beli-6/2019/G19P34	PQ561851
RVC/pig-wt/GER/BE137/2022/G8P21	PQ561852
RVC/pig-wt/GER/BE33/2022/G5	PQ561853
RVC/pig-wt/GER/BE34/2022/G6P21	PQ561854
RVC/pig-wt/GER/BE35/2022/G6P21	PQ561855
RVC/pig-wt/GER/BE36/2022/G6P21	PQ561856
RVC/pig-wt/GER/BE37/2022/G6P6	PQ561857
RVC/pig-wt/GER/BE38/2022/G6P21	PQ561858
RVC/pig-wt/GER/BE39/2022/G6P21	PQ561859
RVC/pig-wt/GER/BE40/2022/G5P4	PQ561860
RVC/pig-wt/GER/BE41/2022/G6P21	PQ561861
RVC/pig-wt/GER/BE42/2022/G6	PQ561862
RVC/pig-wt/GER/BE43/2022/G5P20	PQ561863
RVC/pig-wt/GER/BE44/2022/G1P33	PQ561864
RVC/pig-wt/GER/BE45/2022/G6P6	PQ561865
RVC/pig-wt/DK/BE46/2022/G6	PQ561866
RVC/pig-wt/GER/BE47/2022/G6P32	PQ561867
RVC/pig-wt/DK/BE48/2022/G5P4	PQ561868
RVC/pig-wt/SK/BE49/2022/G6	PQ561869
RVC/pig-wt/GER/Beli-8/2022/G3P34	PQ561870
RVC/pig-wt/GER/Beli-9/2022/G6P32	PQ561871
RVC/pig-wt/GER/BE50/2022/G6P6	PQ561872
RVC/pig-wt/GER/BE51/2022/G6P21	PQ561873
RVC/pig-wt/GER/BE52/2022/G1	PQ561874
RVC/pig-wt/GER/BE53/2022/G6P21	PQ561875
RVC/pig-wt/GER/BE54/2022/G6P21	PQ561876

RVC/pig-wt/GER/BE55/2022/G6P21	PQ561877
RVC/pig-wt/GER/BE56/2022/G6	PQ561878
RVC/pig-wt/GER/BE57/2022/G6P21	PQ561879
RVC/pig-wt/GER/BE58/2022/G6P21	PQ561880
RVC/pig-wt/GER/BE59/2022/G6P21	PQ561881
RVC/pig-wt/GER/BE60/2022/G1P21	PQ561882
RVC/pig-wt/GER/BE61/2022/G6P21	PQ561883
RVC/pig-wt/GER/BE62/2022/G6P21	PQ561884
RVC/pig-wt/GER/BE63/2023/G1	PQ561885
RVC/pig-wt/GER/BE135/2023/G6	PQ561886
RVC/pig-wt/GER/BE64/2023/G6	PQ561887
RVC/pig-wt/GER/BE65/2023/G6P21	PQ561888
RVC/pig-wt/GER/BE66/2023/G3	PQ561889
RVC/pig-wt/GER/BE67/2023/G1	PQ561890
RVC/pig-wt/GER/BE68/2023/G1	PQ561891
RVC/pig-wt/GER/BE69/2023/G6P21	PQ561892
RVC/pig-wt/GER/Beli-10/2023/G5P36	PQ561893
RVC/pig-wt/GER/BE70/2023/G6P21	PQ561894
RVC/pig-wt/GER/Beli-11/2023/G17P35	PQ561895
RVC/pig-wt/GER/BE71/2023/G6P21	PQ561896
RVC/pig-wt/GER/BE72/2023/G6P21	PQ561897
RVC/pig-wt/GER/Beli-12/2023/G5P38	PQ561898
RVC/pig-wt/GER/BE73/2023/G6P21	PQ561899
RVC/pig-wt/GER/BE74/2023/G6P21	PQ561900
RVC/pig-wt/GER/BE75/2023/G6P21	PQ561901
RVC/pig-wt/GER/BE76/2023/G6	PQ561902
RVC/pig-wt/GER/BE77/2023/G5	PQ561903
RVC/pig-wt/SK/BE78/2023/G9P4	PQ561904
RVC/pig-wt/GER/BE79/2023/G6	PQ561905
RVC/pig-wt/GER/BE80/2023/G6	PQ561906
RVC/pig-wt/GER/BE81/2023/G6	PQ561907
RVC/pig-wt/GER/BE82/2023/G6	PQ561908
RVC/pig-wt/GER/BE83/2023/G5P21	PQ561909
RVC/pig-wt/GER/BE84/2023/G6	PQ561910
RVC/pig-wt/GER/BE85/2023/G6	PQ561911
RVC/pig-wt/GER/BE86/2023/G6P21	PQ561912
RVC/pig-wt/GER/BE87/2023/G6P21	PQ561913
RVC/pig-wt/AT/BE88/2023/G6P21	PQ561914
RVC/pig-wt/AT/BE89/2023/G1	PQ561915
RVC/pig-wt/AT/BE90/2023/G6P21	PQ561916
RVC/pig-wt/AT/BE91/2023/G6P21	PQ561917
RVC/pig-wt/AT/Beli-14/2023/G18	PQ561918
RVC/pig-wt/AT/BE92/2023/G6	PQ561919
RVC/pig-wt/AT/BE93/2023/G6	PQ561920
RVC/pig-wt/AT/BE94/2023/G6	PQ561921
RVC/pig-wt/AT/BE95/2023/G1	PQ561922
RVC/pig-wt/AT/BE96/2023/G6P21	PQ561923
RVC/pig-wt/AT/BE97/2023/G6P21	PQ561924
RVC/pig-wt/AT/BE98/2023/G6P21	PQ561925
RVC/pig-wt/AT/BE99/2023/G1	PQ561926
RVC/pig-wt/AT/BE100/2023/G1	PQ561927
RVC/pig-wt/GER/Beli-15/2023/G10P37	PQ561928
RVC/pig-wt/GER/BE101/2023/G6	PQ561929
RVC/pig-wt/GER/BE102/2023/G6	PQ561930
RVC/pig-wt/GER/BE103/2023/G1	PQ561931
RVC/pig-wt/GER/BE104/2023/G1P21	PQ561932
RVC/pig-wt/GER/Beli-13/2023/G6P1	PQ561933
RVC/pig-wt/GER/BE105/2023/G1	PQ561934
RVC/pig-wt/GER/BE106/2023/G8	PQ561935
RVC/pig-wt/GER/BE107/2023/G6	PQ561936
RVC/pig-wt/GER/BE108/2023/G6	PQ561937
RVC/pig-wt/GER/BE109/2023/G6P21	PQ561938
RVC/pig-wt/NL/BE110/2024/G6P21	PQ561939
RVC/pig-wt/GER/BE111/2024/G6P21	PQ561940

RVC/pig-wt/NL/BE112/2024/G6P5	PQ561941
RVC/pig-wt/GER/BE113/2024/G6P5	PQ561942
RVC/pig-wt/BG/Beli-16/2024/G6P30	PQ561943
RVC/pig-wt/GER/BE114/2024/G6P21	PQ561944
RVC/pig-wt/GER/BE115/2024/G6P21	PQ561945
RVC/pig-wt/GER/BE116/2024/G6	PQ561946
RVC/pig-wt/GER/BE117/2024/G6	PQ561947
RVC/pig-wt/NL/BE118/2024/G6P4	PQ561948
RVC/pig-wt/GER/BE119/2024/G1P21	PQ561949
RVC/pig-wt/GER/BE120/2024/G6P6	PQ561950
RVC/pig-wt/GER/BE131/2017/G6P21	PQ606076

Table S1. List of the nucleotide sequences for VP7 (G-type) obtained in this study with associated Accession-Numbers from the NCBI database.

**Strain name (P-type)**	**NCBI-Accession-Number**
RVC/pig-wt/GER/BE1/2016/G6P21	PQ561951
RVC/pig-wt/GER/BE2/2016/G5P4	PQ561952
RVC/pig-wt/GER/BE3/2016/G6P21	PQ561953
RVC/pig-wt/GER/BE4/2017/G5P6	PQ561954
RVC/pig-wt/GER/Beli-1/2017/G6P31	PQ561955
RVC/pig-wt/GER/BE8/2017/G6P6	PQ561956
RVC/pig-wt/GER/BE10/2017/G6P21	PQ561957
RVC/pig-wt/GER/BE11/2017/G6P21	PQ561958
RVC/pig-wt/GER/BE14/2017/G6P21	PQ561959
RVC/pig-wt/GER/BE15/2017/G6P21	PQ561960
RVC/pig-wt/GER/BE17/2017/G6P21	PQ561961
RVC/pig-wt/GER/BE130/2017/P21	PQ561962
RVC/pig-wt/GER/BE131/2017/G6P21	PQ561963
RVC/pig-wt/GER/BE18/2017/G6P21	PQ561964
RVC/pig-wt/GER/BE20/2017/G6P21	PQ561965
RVC/pig-wt/GER/BE121/2017/G6P31	PQ561966
RVC/pig-wt/GER/BE21/2018/G6P21	PQ561967
RVC/pig-wt/GER/BE22/2018/G6P21	PQ561968
RVC/pig-wt/GER/BE25/2018/G6P21	PQ561969
RVC/pig-wt/GER/BE26/2018/G6P5	PQ561970
RVC/pig-wt/GER/BE27/2018/G6P21	PQ561971
RVC/pig-wt/GER/Beli-4/2018/G6P39	PQ561972
RVC/pig-wt/GER/BE129/2018/P4	PQ561973
RVC/pig-wt/GER/Beli-5/2018/G1P33	PQ561974
RVC/pig-wt/GER/BE31/2018/G6P21	PQ561975
RVC/pig-wt/GER/Beli-6/2019/G19P34	PQ561976
RVC/pig-wt/GER/BE125/2019/P21	PQ561977
RVC/pig-wt/GER/BE137/2022/G8P21	PQ561978
RVC/pig-wt/GER/BE34/2022/G6P21	PQ561979
RVC/pig-wt/GER/BE35/2022/G6P21	PQ561980
RVC/pig-wt/GER/BE36/2022/G6P21	PQ561981
RVC/pig-wt/GER/BE127/2022/P6	PQ561982
RVC/pig-wt/GER/BE38/2022/G6P21	PQ561983
RVC/pig-wt/GER/BE39/2022/G6P21	PQ561984
RVC/pig-wt/GER/BE40/2022/G5P4	PQ561985
RVC/pig-wt/GER/BE41/2022/G6P21	PQ561986
RVC/pig-wt/GER/BE43/2022/G5P20	PQ561987
RVC/pig-wt/GER/BE44/2022/G1P33	PQ561988
RVC/pig-wt/GER/BE128/2022/P33	PQ561989
RVC/pig-wt/GER/BE45/2022/G6P6	PQ561990
RVC/pig-wt/GER/BE47/2022/G6P32	PQ561991
RVC/pig-wt/DK/BE48/2022/G5P4	PQ561992

RVC/pig-wt/GER/Beli-8/2022/G3P34	PQ561993
RVC/pig-wt/GER/Beli-9/2022/G6P32	PQ561994
RVC/pig-wt/GER/BE50/2022/G6P6	PQ561995
RVC/pig-wt/GER/BE51/2022/G6P21	PQ561996
RVC/pig-wt/GER/BE53/2022/G6P21	PQ561997
RVC/pig-wt/GER/BE54/2022/G6P21	PQ561998
RVC/pig-wt/GER/BE55/2022/G6P21	PQ561999
RVC/pig-wt/GER/BE57/2022/G6P21	PQ562000
RVC/pig-wt/GER/BE58/2022/G6P21	PQ562001
RVC/pig-wt/GER/BE59/2022/G6P21	PQ562002
RVC/pig-wt/GER/BE60/2022/G1P21	PQ562003
RVC/pig-wt/GER/BE61/2022/G6P21	PQ562004
RVC/pig-wt/GER/BE62/2022/G6P21	PQ562005
RVC/pig-wt/GER/BE65/2023/G6P21	PQ562006
RVC/pig-wt/GER/BE121/2023/P20	PQ562007
RVC/pig-wt/GER/BE69/2023/G6P21	PQ562008
RVC/pig-wt/GER/Beli-10/2023/G5P36	PQ562009
RVC/pig-wt/GER/BE122/2023/P21	PQ562010
RVC/pig-wt/GER/BE123/2023/P6	PQ562011
RVC/pig-wt/GER/BE124/2023/P21	PQ562012
RVC/pig-wt/GER/Beli-11/2023/G17P35	PQ562013
RVC/pig-wt/GER/BE71/2023/G6P21	PQ562014
RVC/pig-wt/GER/BE72/2023/G6P21	PQ562015
RVC/pig-wt/GER/Beli-12/2023/G5P38	PQ562016
RVC/pig-wt/GER/BE73/2023/G6P21	PQ562017
RVC/pig-wt/GER/BE74/2023/G6P21	PQ562018
RVC/pig-wt/GER/BE75/2023/G6P21	PQ562019
RVC/pig-wt/SK/BE78/2023/G9P4	PQ562020
RVC/pig-wt/GER/BE83/2023/G5P21	PQ562021
RVC/pig-wt/GER/BE126/2023/P21	PQ562022
RVC/pig-wt/GER/BE86/2023/G6P21	PQ562023
RVC/pig-wt/GER/BE87/2023/G6P21	PQ562024
RVC/pig-wt/AT/BE88/2023/G6P21	PQ562025
RVC/pig-wt/AT/BE90/2023/G6P21	PQ562026
RVC/pig-wt/AT/BE91/2023/G6P21	PQ562027
RVC/pig-wt/AT/BE96/2023/G6P21	PQ562028
RVC/pig-wt/AT/BE97/2023/G6P21	PQ562029
RVC/pig-wt/AT/BE98/2023/G6P21	PQ562030
RVC/pig-wt/GER/Beli-15/2023/G10P37	PQ562031
RVC/pig-wt/GER/BE104/2023/G1P21	PQ562032
RVC/pig-wt/GER/Beli-13/2023/G6P1	PQ562033
RVC/pig-wt/GER/BE132/2023/P21	PQ562034
RVC/pig-wt/SK/BE133/2023/P21	PQ562035
RVC/pig-wt/GER/BE134/2023/P35	PQ562036
RVC/pig-wt/GER/BE109/2023/G6P21	PQ562037
RVC/pig-wt/NL/BE110/2024/G6P21	PQ562038
RVC/pig-wt/GER/BE111/2024/G6P21	PQ562039
RVC/pig-wt/NL/BE112/2024/G6P5	PQ562040
RVC/pig-wt/GER/BE113/2024/G6P5	PQ562041
RVC/pig-wt/BG/Beli-16/2024/G6P30	PQ562042
RVC/pig-wt/GER/BE114/2024/G6P21	PQ562043
RVC/pig-wt/GER/BE115/2024/G6P21	PQ562044
RVC/pig-wt/NL/BE118/2024/G6P4	PQ562045
RVC/pig-wt/GER/BE119/2024/G1P21	PQ562046
RVC/pig-wt/GER/BE120/2024/G6P6	PQ562047

Table S2. List of the nucleotide sequences for VP4 (P-type) obtained in this study with associated Accession-Numbers from the NCBI database.
